# Total Vaginal Hysterectomy Can Provide a Feasible Surgical Approach for Benign Conditions: A Study on Enlarged Diffuse Uterine Adenomyosis from Romania

**DOI:** 10.3390/healthcare14121677

**Published:** 2026-06-12

**Authors:** Mihnea Nicodin, Laura Nicodin-Tigoianu, Anca Popescu, Mariam Dalaty, Diana Badiu, Lucian Cristian Petcu, Ovidiu Nicodin, Cristian Delcea, Nicolae Suciu

**Affiliations:** 1 Doctoral School of Medicine, “Carol Davila” University of Medicine and Pharmacy, 8, Eroii Sanit Blvd, 050474 Bucharest, Romania; 2 “Carol Davila” Central Military Emergency University Hospital, 134, Plevnei Street, Sector 1, 010825 Bucharest, Romania; 3 Faculty of Medicine, Titu Maiorescu University, 67 A, Gheorghe Petrascu Street, Sector 3, 031593 Bucharest, Romania; 4 Alessandrescu Rusescu National Institute for the Health of Mother and Child, 38-54, Gh. Polizu Street, 020395 Bucharest, Romania; 5 Doctoral School of Medicine and Pharmacy, “George Emil Palade” University of Medicine, Pharmacy, Science, and Technology of Targu Mures, 38, Gh. Marinescu Street, 540142 Targu Mures, Romania; 6 Faculty of Medicine, “Ovidius” University from Constanta, 1, University Str., 900470 Constanta, Romania; 7 Doctoral School of Medicine, “Ovidius” University from Constanta, 1, University Str., 900470 Constanta, Romania; 8 Multidisciplinary Doctoral School, Vasile Goldis Western University of Arad, 94, Bvd. Revolution, 310025 Arad, Romania

**Keywords:** diffuse adenomyosis, enlarged uterus, vaginal hysterectomy, operative time, morcellation

## Abstract

**Background:** The total vaginal hysterectomy (TVH) technique has been performed for many gynecological surgeries but is less used than total abdominal hysterectomy (TAH). The purpose of this study was to analyze the outcomes in patients who underwent either TVH or TAH for enlarged diffuse uterine adenomyosis (ADS). **Methods:** In this retrospective study, 160 hysterectomies with bilateral adnexectomy were scheduled for premenopausal women with diffuse ADS between 2020 and 2024 at a tertiary care center in Romania. The diagnosis was established based on clinical symptomatology, color Doppler transvaginal ultrasound, transabdominal ultrasonography and magnetic resonance imaging with histopathological confirmation of diffuse uterine ADS post-operatively from the hysterectomy specimen. All surgical procedures were performed by the same surgeon. The patients were divided into two groups: the TVH group (*n* = 80) and the TAH group (*n* = 80). Patient characteristics, including age, uterine weight, body mass index (BMI), parity, operative time, intraoperative blood loss, length of hospital stay, medical history, surgical history, intra- and postoperative complications, and the presence of adhesions, were evaluated. **Results:** No differences were observed between the two groups with regard to uterine weight, BMI, or parity. However, the TVH group was associated with significantly younger age, shorter operative time, lower intraoperative blood loss, and a reduced length of hospital stay (*p* < 0.001). Additionally, both medical and surgical histories were more frequently observed in the TAH group than in the TVH group. However, intra- (i.e., 3.75%) and postoperative complications (i.e., 13.75% vs. 3.75%) were slightly more frequent in the TAH group, as well as adhesions (i.e., 17.50% vs. 8.75%). Uterine hemisection, tactical myomectomy, or morcellation were frequently employed in the TVH group, being required in 77.5% of cases. **Conclusions:** In our cohort of patients with enlarged diffuse uterine ADS, TVH was associated with younger age, shorter operative time, reduced intraoperative blood loss, and a shorter length hospital stay compared with TAH, without an observed increase in perioperative complications. These results support the use of TVH as a feasible surgical approach for selected enlarged diffuse uterine ADS.

## 1. Introduction

Adenomyosis (ADS) is a benign gynecological condition characterized by the presence of endometrial glands within the myometrium, frequently associated with hyperplasia with a prevalence of 28.2% in hysterectomy specimens [1,2]. Different symptoms like abnormal uterine bleeding, dysmenorrhea and uterine tenderness caused by ADS can lead to progressive uterine enlargement [3]. However, most gynecological symptoms, like abnormal uterine bleeding, dysmenorrhea or uterine tenderness caused by ADS, can be associated with progressive uterine enlargement symptoms [3].

The reliability of establishing a definitive pre-operative diagnosis of ADS based solely on clinical symptomatology still remains debated [4]. Currently, there is no standardized or universally accepted treatment protocol for ADS [5]. Although medical therapies, like anti-estrogens, gonadotropin-releasing hormone agonists, and levonorgestrel-releasing intrauterine systems, can provide symptom relief in this estrogen-dependent disorder, hysterectomy continues to be the only definitive treatment [6].

The exact cause of ADS remains uncertain, and advances in pelvic imaging, particularly magnetic resonance imaging (MRI), have now made it possible to identify the condition more accurately. However, because ADS has rarely been diagnosed before hysterectomy, hormonal therapies that aim to suppress endometrial cell proliferation still show robust clinical evidence. For over a century, hysterectomy has served as both the primary diagnostic and finally therapeutic approach to uterine ADS [7].

The decision to perform a hysterectomy, whether abdominally or vaginally, often depends not only on the clinical indication but also on the surgeon’s expertise. In a large comparative study involving total vaginal hysterectomy (TVH) in patients with ADS, Furuhashi et al. [7] reported that women with ADS faced a significantly higher risk of bladder injury, although blood loss and operative time did not differ once uterine weight was considered. The underlying mechanism responsible for the increased risk of bladder injury remains uncertain, although it may be associated with the difficulty in identifying the supravaginal septum intraoperatively [8]. However, in many cases, the diagnosis of ADS was established retrospectively, based on histopathological examination of the hysterectomy specimen, rather than pre-operatively [8].

Different ultrasound features of ADS have been described in the literature, including the “question mark” sign, heterogeneous myometrium, uterine asymmetry, subendometrial thickening, or uterine enlargement [9].

On MRI, the most important diagnostic criterion appears to be junctional zone irregularity [10], followed by focal or diffuse thickening of the junctional zone [11,12]. Transvaginal, hysteroscopic, or laparoscopic biopsy techniques could also support diagnosis by providing histological confirmation [13]. However, the true prevalence of this common benign condition remains unclear, as most available data are based on its incidence in hysterectomy specimens [14].

The objective of this study was to evaluate outcomes in patients from our institutional cohort who underwent either TVH or total abdominal hysterectomy (TAH) for enlarged diffuse uterine ADS with a uterine weight between 250 and 300 g.

## 2. Materials and Methods

### 2.1. Study Design and Participants

A total of 160 patients were included in this retrospective, non-randomized cohort study conducted over a four-year period, between 1 March 2020, and 30 July 2024, in which no multivariable adjustment was performed to account for baseline differences between groups, including age, medical history, and prior surgical history. The study population consisted of patients scheduled to undergo hysterectomy with bilateral adnexectomy for enlarged diffuse uterine ADS in the Department of Gynecology at the “Carol Davila” Central Military Emergency University Hospital, Bucharest, a tertiary care center in Romania. All patients were in the late premenopausal stage, based on age and symptoms, and were divided into two groups: the TVH group (*n* = 80) and the TAH group (*n* = 80). The inclusion criteria for the TVH group comprised patients of whom approximately 12.5% were nulliparous and 35% were primiparous. Selection of the vaginal route was primarily based on uterine mobility, as assessed during the preoperative examination. Additional factors favoring TVH included a limited history of previous surgical interventions, with cesarean delivery reported in 13.75% of cases, as well as adequate vaginal length and width to ensure optimal surgical exposure. Preoperative route selection also considered the anticipated feasibility of vaginal uterine extraction, clinical suspicion of extensive pelvic adhesions, and the expected need for uterine reduction techniques such as hemisection, tactical myomectomy, or morcellation. Patients with reduced uterine mobility, more complex surgical history, suspected difficult pelvic access, or anticipated technical limitations were more likely to undergo TAH. Nevertheless, surgeon experience represented the most important determinant in the decision to attempt TVH, reflecting routine clinical practice in complex hysterectomy cases. This study was conducted and reported in accordance with the STROBE guidelines for observational studies.

Data collected included patient age, uterine weight, body mass index (BMI), parity, operative time, intraoperative blood loss, length of hospital stay, medical history, surgical history, intra- and postoperative complications, and the presence of adhesions.

Patients were excluded if they were admitted for uterine prolapse, cervical dysplasia, pelvic inflammatory disease, or any malignancy. All patients meeting the inclusion criteria, namely, diffuse ADS of the uterus with a uterine weight between 250 and 300 g, with or without additional pathologies such as leiomyoma, and abnormal uterine bleeding as the primary symptom, were entered in the study. Besides the most persistent symptom, patients also presented with very rare periods, hot flashes, night sweats, mood swings, and vaginal dryness in the last four years.

Patients were diagnosed based on clinical symptomatology, color Doppler transvaginal ultrasound findings, and MRI. MRI diagnosis relied primarily on junctional zone thickening and irregularity. Transvaginal ultrasonographic features suggestive of ADS included heterogeneous myometrium, asymmetric myometrial thickening, globular uterine enlargement, subendometrial echogenic linear striations, and indistinct endometrial-myometrial junctions, according to Morphological Uterus Sonographic Assessment criteria. In addition, transabdominal ultrasonography using a 3.5 MHz probe was performed preoperatively for uterine size and volume measurements. Two views were obtained, longitudinal and transverse. In the longitudinal view, the longitudinal measurement was taken from the highest fundal point in the midline to the corresponding midline cervico-uterine junction (uterine length). In the same view, the anteroposterior measurement (uterine depth) was obtained perpendicular to this plane at the widest fundal dimension. In the transverse view, the largest transverse uterine diameter was measured at the level of the tubal insertion. Uterine volume was calculated using the prolate ellipsoid equation [15]. In our cases, an endometrial biopsy was systematically performed on all patients preoperatively to rule out preinvasive or invasive endometrial pathology. This practice is essential, especially before performing TVH, as different reduction size techniques are employed, such as hemisection, tactical myomectomy, or morcellation. Histopathological confirmation of diffuse uterine ADS was obtained postoperatively from the hysterectomy specimen, according to our hospital protocol. All patients underwent preoperative imaging evaluation by a single experienced operator.

All adhesions were assessed intraoperatively. A urinary catheter was placed pre-operatively in all patients. For each patient, a prophylactic intravenous cephalosporin antibiotic was administered before the procedure. All surgical procedures were carried out by the first author. Written informed consent was obtained from all participants prior to enrollment, after they were thoroughly informed about the surgical procedures, associated risks, and potential limitations of both TAH and TVH. However, our indications of TVH to convert at TAH were: massive hemorrhage, excessive adhesions, injury to surrounding structures, or issues of fallopian tubes which require broader access. Below we describe the TVH and TAH techniques used in our department.

### 2.2. TVH Technique

The patient was placed in dorsal lithotomy location, with the buttocks positioned beyond the edge of the table. A comprehensive pelvic examination was then performed under anesthesia to confirm the suitability of the vaginal approach for hysterectomy, with particular attention to the pelvic arch configuration and vaginal capacity. A traction test assessed uterine mobility, which represented a key component in the decision to proceed with the vaginal approach. The operative field was prepared using povidone-iodine solution. Then, the cervix was grasped using two clamps for obtaining optimal traction, and the cervicovaginal junction was identified. Hydrodissection was achieved by infiltrating 20–50 mL of saline solution into the cervical area designated for incision. The anterior vaginal wall was then incised through the full thickness of the vaginal epithelium at its junction with the cervix. Subsequently, the vaginal wall was retracted and the bladder was gently displaced caudally. After identification of the anterior peritoneal fold, it was grasped, elevated, and incised. A speculum was used to cover the bladder during the dissection. The posterior part usually began with a transverse incision extending from the 4 to 8 o’clock position. The posterior vaginal edge was grasped with two clamps and carefully dissected until the posterior peritoneal fold was reached. The peritoneum was then incised with scissors, confirming entry into the posterior cul-de-sac. A long vaginal speculum was placed in the posterior pouch to provide adequate retraction. The uterosacral ligament was clamped, transected, and ligated, and the cardinal ligament was managed in a similar manner. On each side, both ligaments were typically clamped and ligated together. The same procedure was subsequently performed on the contralateral side. In cases where anterior peritoneal access was not feasible due to abnormal vesico-uterine adhesions, further uterine mobilization was achieved after clamping, transecting, and ligating the subsequent pedicles. The uterine vessels were subsequently identified and clamped between the anterior and posterior peritoneal reflections using a Heaney clamp, after which they were transected and secured with transfixion ligatures. Progressive bilateral clamping of the uterine artery was then performed in a cranial direction using the same technique. The utero-ovarian vessels, fallopian tube, and round ligament were then double-clamped, transected, and ligated, thereby completing the disconnection of the uterus and concluding the hysterectomy.

Following uterine removal, a routine inspection of the adnexa was performed, according to the specific case, by applying lateral and downward traction to the utero-ovarian pedicle clamps. Once the fallopian tubes and ovaries were visualized, they were grasped with a clamp, and gentle traction was applied to the adnexa. Subsequently, a Heaney clamp was placed lateral to the utero-ovarian pedicle and advanced superiorly above the tube and ovary in order to clamp the infundibulo-pelvic ligament. A resorbable suture was then placed around the pedicle at the level of the Heaney clamp, and the pedicle was double-clamped and ligated to ensure additional security. In addition, a portable electrosurgical vessel-sealing device was used to facilitate adnexal excision. Hemostasis was systematically assessed, beginning at the cervicovaginal pedicle and proceeding in a clockwise direction.

The vaginal vault was subsequently closed by approximating the anterior and posterior vaginal walls using interrupted absorbable “X” sutures. A peritoneal drain was placed in both the TVH and TAH groups in accordance with our department’s standard surgical protocol for hysterectomy. In the TVH group, the drain was introduced through the vaginal cuff into the abdominal cavity, whereas in the TAH group it was inserted via the abdominal route. Both drains were placed in order to prevent the accumulation of blood and fluids, reducing the risks of infection, fever, and severe pain, acting as a safety measure. In both groups, the drain was routinely removed 48 h postoperatively.

Vaginal packing with gauze soaked in povidone-iodine was performed to monitor postoperative bleeding and to facilitate hemostasis through compression. Cystourethroscopy was also performed at the end of the procedure in cases where inadvertent bladder injury was suspected, in order to confirm the integrity of the bladder and ureters.

### 2.3. TAH Technique

The patient was positioned in the Trendelenburg position after preparation of the surgical field. A vesico-urethral catheter was inserted, with continuous monitoring of urine output and characteristics. The abdominal approach was performed via either a Pfannenstiel or Maylard incision, depending on the specific requirements of the case. For the Maylard incision, the aponeurosis was first incised, followed by transection of the rectus abdominis muscles, with subsequent clamping, transection, and ligation of the inferior epigastric vessels. Upon entry into the peritoneal cavity, the median umbilical ligament was clamped, transected, and ligated, with its lower portion used to facilitate exposure of the surgical field by providing traction on the urinary bladder. A systematic inspection of the peritoneal cavity was then performed, with adhesiolysis carried out as necessary. For effective uterine traction, two Pean-type forceps were applied bilaterally to the uterine horns, encompassing the insertions of the round ligament, utero-ovarian ligament, and fallopian tube. The round ligaments were subsequently clamped with Kocher forceps, transected, and ligated bilaterally.

Subsequently, the posterior leaf of the broad ligament was incised to allow retroperitoneal access to the lumbo-ovarian ligament. The infundibulopelvic ligament was then clamped using J.L. Faure-type forceps, transected, and double-ligated with 2-0 Vicryl absorbable suture. Sectioning of the broad ligament sheets was continued toward the uterine vicinity, followed by detachment of the appendix. Uni- or bilateral adnexectomy was then performed. The utero-ovarian ligament was clamped with J.L. Faure forceps, transected, and ligated using 0 or 1 Vicryl absorbable suture.

The operative time was recorded from the initial incision until the completion of the procedure. The surgical nurse routinely estimated the amount of intraoperative blood loss for both techniques to make the same estimate of the amount of blood lost and to avoid measurement differences, which was calculated based on the number of soaked mops. As a rough guide, a quarter-soaked mop was considered to contain approximately 20 mL of blood, a half-soaked mop 40 mL, and a fully soaked mop 80 mL. The specimen was weighed immediately after surgery. Hospital stay was defined as the total number of days the patient remained hospitalized.

### 2.4. Uterine Reduction Techniques

The subsequent surgical steps following TVH were determined by the size and characteristics of the uterine mass. In most cases, uterine hemisection, tactical myomectomy, or morcellation was employed, as described below.

Uterine hemisection involved dividing the uterus along the sagittal plane, beginning at the cervix and proceeding cranially, with the incision carefully maintained within the uterine cavity to prevent lateral deviation and minimize hemorrhage. Upon completion of the hemisection, one half of the specimen was temporarily retained within the pelvic cavity, thereby allowing safe exposure of the contralateral side for subsequent ligation and dissection.

Tactical myomectomy was primarily used when central nodules were present in the uterine mass. Nodules were identified using Tirre-Balle or Museaux forceps and removed either digitally or during intramyometrial reduction or morcellation. Morcellation was additionally employed to sequentially excise myometrial fragments as needed.

### 2.5. Postoperative Follow-Up

Patients were managed postoperatively according to a standardized pain management protocol, which included two doses of intravenous meperidine, 50 mg every 4 h, followed by acetaminophen, 325 mg every 6 h. Patients were discharged once they were able to control pain with oral medication alone, tolerate a soft diet, void independently, and maintain normal bowel function.

Intra- and postoperative complications were classified according to standard classification. Intraoperative complications were documented, including depolishing of a large intestinal loop and inadvertent cystostomy requiring cystorrhaphy. Postoperative complications were recorded descriptively and categorized by type and were recorded as follows: urinary tract infection (UTI), wound seroma, dynamic ileus and secondary anemia. All patients were scheduled for follow-up two weeks after surgery, and final pathologic findings of the specimen were compared with the preoperative ultrasound, endometrial biopsy, and MRI results.

### 2.6. Statistical Analysis

Statistical analyses were conducted using IBM SPSS Statistics version 23. Continuous variables are summarized in Table 1 using standard measures of dispersion and central tendency, while categorical variables are presented as percentages for each category in Table 2.

The normality of continuous variables was assessed using the Kolmogorov–Smirnov test. For hypothesis testing, the Independent Samples *t*-test, Mann–Whitney U test, and median test were applied, with a significance level (α) set at 0.05. The Kolmogorov–Smirnov test indicated that the normality assumption was satisfied only for age (*p* > 0.05), whereas most other group variables violated this assumption (*p* < 0.05). Accordingly, nonparametric methods (i.e., Mann–Whitney U and median tests) were employed for the respective analyses.

## 3. Results

**Figure 1:**
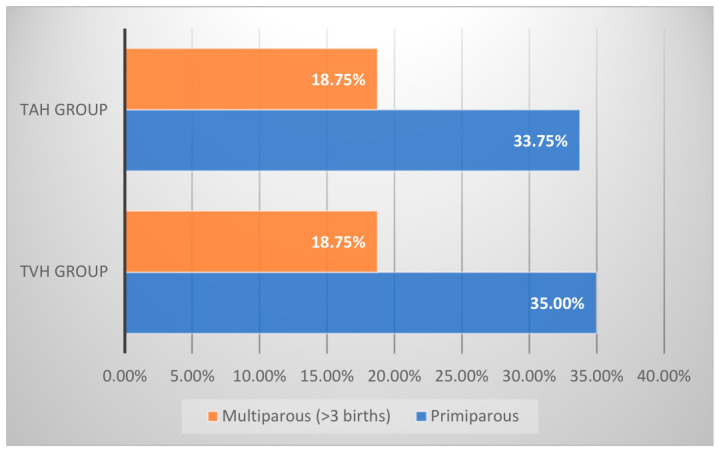
Distribution of patients according to surgical approach.

A total of 160 patients underwent either TVH or TAH during the study period. The most common indications were late premenopausal-stage women with diffuse ADS and a uterine weight between 250 and 300 g. However, the selection of allocated patients for TVH vs. TAH is represented in Figure 1.

TVH with bilateral adnexectomy was successfully performed in all patients (100%), whereas TAH with bilateral adnexectomy was achieved only in 77 patients (96.25%). The other patients had previously undergone adnexectomy. From the last group, only 1 patient (1.25%) had unilateral salpingectomy, which did not affect our results.

**Figure 2:**
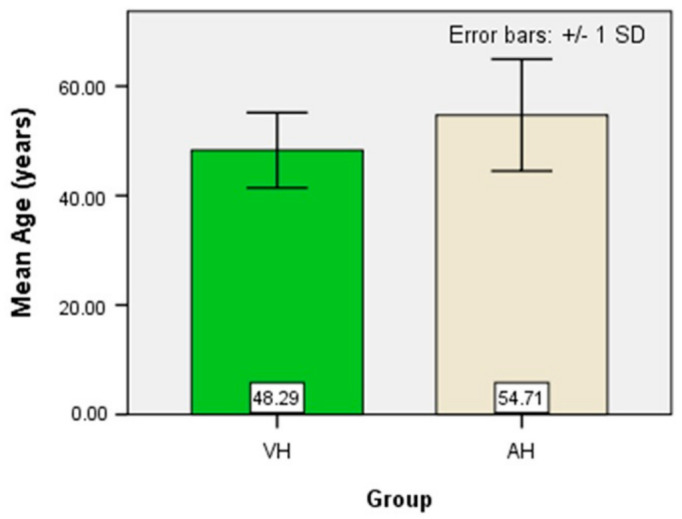
Comparison of mean age between the TVH and TAH groups.

The main clinical characteristics, with measures of dispersion for continuous variables, for patients undergoing TVH and TAH are presented in Table 1. Notably, a statistically significant difference was observed between the mean ages of the two groups: TVH (48.29 ± 6.88 years) and TAH (54.71 ± 10.21 years; t = −4.667, df = 138.475, *p* < 0.001, α = 0.05 with 95% CI) (Figure 2).

For the variable of uterine weight, the nonparametric Mann–Whitney U test indicated no statistically significant difference between the TVH group (median = 278.00, IQR = 26.5, mean rank = 77.93) and the TAH group (median = 278.00, IQR = 28.75, mean rank = 83.07; U = 2994.50, z = −0.702, *p* = 0.483, α = 0.05). This finding was further confirmed by the median test, which also showed no statistically significant difference (chi-square = 0.025, df = 1, *p* = 0.874, α = 0.05) (Table 1).

The nonparametric Mann–Whitney U test for the BMI variable revealed no statistically significant difference between the TVH group (median = 29.35, IQR = 6.23, mean rank = 79.07) and TAH group (median = 29.25, IQR = 6.03, mean rank = 81.93; U = 3085.50, z = −0.391, *p* = 0.696, α = 0.05). The median test yielded consistent results, indicating no significant difference between the groups (chi-square = 0.025, df = 1, *p* = 0.874, α = 0.05).

Similar findings were observed for parity, with no statistically significant difference between the TVH group (median = 2.00, IQR = 1.00, mean rank = 80.16) and TAH group (median = 2.00, IQR = 1.00, mean rank = 80.84; U = 3172.50, z = −0.098, *p* = 0.098, α = 0.05). The median test confirmed the absence of significant differences between the groups (chi-square = 0, df = 1, *p* = 1.000, α = 0.05).

Statistically significant differences were observed for operative time, with lower values in the TVH group (median = 55.00, IQR = 20.5, mean rank = 56.11) compared to the TAH group (median = 82.26, IQR = 48.25, mean rank = 104.89; U = 1249.00, z = −6.659, *p* < 0.001, α = 0.05). These findings were confirmed by the median test (chi-square = 25.600, df = 1, *p* < 0.001, α = 0.05).

Similarly, for intraoperative blood loss, the Mann–Whitney U test revealed statistically significant differences between the TVH group (median = 114.50, IQR = 48.75, mean rank = 61.28) and the TAH group (median = 135.00, IQR = 40, mean rank = 99.73; U = 1662.00, z = −5.254, *p* < 0.001, α = 0.05), which was corroborated by the median test (chi-square = 13.227, df = 1, *p* < 0.001, α = 0.05).

Regarding hospital stay, statistically significant differences was noted, with shorter stays in the TVH group (median = 4.00, IQR = 2.00, mean rank = 54.30) compared to the TAH group (median = 5.00, IQR = 2.00, mean rank = 106.70; U = 1104.00, z = −7.391, *p* < 0.001, α = 0.05). These results were consistent with the median test (chi-square = 28.900, df = 1, *p* < 0.001, α = 0.05).

A review of medical history indicated that comorbidities were more prevalent in the TAH group, with 13.75% of patients presenting secondary anemia, 1.25% with type 2 diabetes, 6.25% with hypertension, 2.50% with dyslipidemia, 7.50% with hypothyroidism, 1.25% with both secondary anemia and hypothyroidism, and 1.25% with anxiety syndrome. In comparison, the TVH group included 12.50% with secondary anemia, 6.25% with hypertension, 2.50% with dyslipidemia, 7.50% with hypothyroidism, and 1.25% with rheumatoid arthritis.

Analysis of surgical history revealed similar patterns. In the TAH group, 10.00% had undergone appendectomy, 3.75% bilateral adnexectomy, 35.00% cesarean section, 1.25% appendectomy combined with cholecystectomy, 1.25% gastric sleeve, 1.25% unilateral ovarian cystectomy, and 1.25% unilateral salpingectomy. In the TVH group, 12.50% had appendectomy, 13.75% cesarean section, and 1.25% appendectomy with cholecystectomy (Table 2).

Intraoperative complications were rare, occurring in only 3.75% of the TAH group, comprising three cases of depolishing of a large intestinal loop. Postoperative complications were slightly more frequent in the TAH group compared to the TVH group. In the TVH group, 3.75% of patients experienced complications, including one case of UTI and two cases of secondary anemia. In the TAH group, 13.75% of patients experienced complications, including three cases of UTI, three cases of wound seroma, one case of dynamic ileus, and four cases of secondary anemia. However, there was a statistically significant difference between the proportion of patients with postoperative complications in the TVH group (p_1_ = 0.0375) compared with the proportion of patients with postoperative complications in the TAH group (p_2_ = 0.1375): *p*-diff = −0.100, z = −2.238, *p*-value = 0.025 < 0.05, 95% CI for *p*-diff: −0.186 to −0.014 (Table 2).

**Figure 3:**
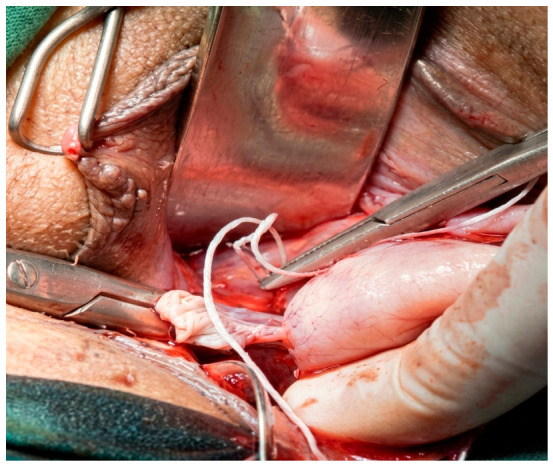
Gross anatomy of the ligature of the lower uterine pedicles.

**Figure 4:**
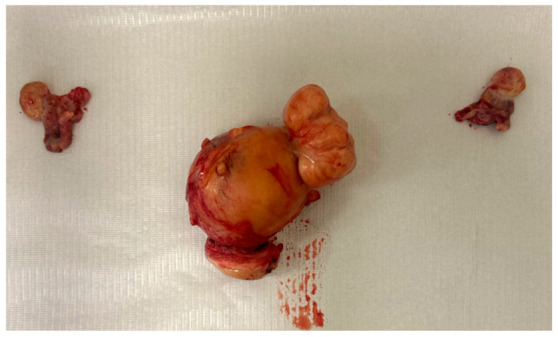
Gross anatomy of the uterus and ovaries excised after TVH.

The planned TVH procedures were successfully completed in all patients without the need for conversion to laparotomy (Figure 3 and Figure 4).

Adhesions were more frequently observed in the TAH group (17.50%) compared to the TVH group (8.75%). In the TVH group, uterine hemisection, tactical myomectomy, and morcellation were employed in the majority of cases (77.5%) to achieve uterine size reduction, whereas intramyometrial reduction and morcellation following cervical amputation were utilized in a smaller proportion of cases (22.5%).

## 4. Discussion

### 4.1. Patients Outcome

This study aimed to compare the characteristics of women with diffuse ADS undergoing either TVH or TAH with bilateral adnexectomy. In our cohort, TVH was associated with feasible perioperative outcomes, including younger age, shorter operative time, reduced estimated blood loss, and shorter hospital stay, without an observed increase in perioperative complications. These findings suggest that TVH may represent a feasible surgical option for selected patients with diffuse ADS and support further investigation in comparative studies. However, the number of patients included in the TVH group was equal to that of the TAH group. This distribution may be explained by the profile of our department, which specializes in gynecological oncology and has extensive experience in minimally invasive surgical approaches, especially VHs. These correlations are consistent with those of previous studies of Benassi et al. [16], which compared the outcomes of the two hysterectomy approaches and reported no significant differences in complications, except for postoperative fever. Similarly, Garry & Hertz [17] had comparable results, noting shorter operative times in favor of TVH. Furthermore, the study of Balakrishnan et al. [18] showed that blood loss during TVH was more reduced than during TAH. However, other studies reported that TVH is effective and can be safely performed even in patients with large, immobile uteri or a history of previous pelvic surgery [19,20].

In the present study, the intraoperative complication rate for TVH was lower than for TAH, similar to the findings reported by Debodinance [21]. Another study reported intraoperative complication rates of 3.2% for TVH vs. 6.5% for TAH [22]. Collectively, these studies indicated that TVH represents a safe and effective surgical approach for benign gynecological conditions. Appropriate patient selection, psychosocial risks and surgeon experience are likely critical factors due to the sensitivity of TVH performance in moderate or enlarged uteri [23].

The most common presenting symptom in our study population was abnormal uterine bleeding, which is frequently observed in this age group [24]. However, in the TVH group, it was seen that patients were younger (48.29 ± 6.88 years vs. 54.71 ± 10.12 years) and had fewer comorbidities compared with the TAH group (57.50% vs. 87.50%). These findings are in accordance with the study of Rivas-Arredondo and contributors. Those results showed a sample of 332 patients with ADS, a median age of 45 years with an interquartile range of 39–50 years after total or sub-TH. About 63.9% of women had a history of cesarean section, and 72.9% were categorized as women with multiple pregnancies. Another 19% of the women had diabetes, and 28.6% had hypertension [24]. Another study sustained that patients with ADS alone had a lower prevalence of comorbidities compared with patients with both ADS and endometriosis, suggesting a different clinical profile [25]. Therefore, while traditionally diagnosed in perimenopausal women, studies showed that patients with symptomatic ADS often present for surgical treatment at a younger age and frequently have fewer comorbidities. However, parity and age at diagnosis may be stratifying factors in future clinical trials on hormone therapy for such patients [26].

Moreover, the abdominal approach was associated with longer hospital stays, higher complication rates, and increased costs. In contrast, TVH offers shorter hospital stays and faster recovery times [27]. Given its lower risk of complications, TVH is generally preferred, highlighting a noticeable change in patient behavior, especially when uterine weight is below 280 g [28]. It is well established that increasing uterine weight reduces preoperative assessment and interpretation of uterine weight before choosing the surgical route [29], in which the condition should be visualized and interpreted in a proper manner preoperatively [30].

Heavier uteri are also associated with greater perioperative blood loss, as the vessels supplying a large uterus tend to be thicker [31]. Another study reported a significant increase in the development of microvessels in patients with ADS [32]. Adenomyotic lesions are highly vascularized, largely due to elevated levels of vascular endothelial growth factor [33]. Therefore, patients with ADS can be expected to experience increased perioperative blood loss and a higher likelihood of requiring postoperative blood transfusion. Further, the American College of Obstetrics and Gynecology recommends the use of cystoscopy in patients with a higher index of suspicion for UTI [34], as the greatest risk of ureteral trauma occurs in proximity to the uterine artery [35].

The study of Mishra et al. [36], including 150 patients, reported lower complication rates, shorter hospital stays, and reduced intraoperative blood loss following total laparoscopic hysterectomy, supporting its role as a minimally invasive alternative to conventional approaches, such as TAH. With advances in surgical techniques and increasing expertise in minimally invasive gynecology, AH is now performed more selectively, while laparoscopic and vaginal approaches are increasingly favored when clinically appropriate. Nevertheless, despite these developments, approximately 600,000 hysterectomies are still performed annually in the United States, with nearly 70% being carried out through an abdominal approach [37].

One study reported increased rates for benign uterine pathology of bladder and ureteral complications for VH in ADS patients [38]. Another study showed that VH is generally safe for ADS, and further, ADS was associated with a higher risk of bladder injury compared with leiomyomas, while operative time and blood loss were similar when adjusted for uterine weight [39]. However, patients undergoing hysterectomy with bilateral adnexectomy for ADS should be counseled about an increased risk of bladder injury and persistent pelvic pain, again referring to Furuhashi’s VH data as key evidence [40]. However, it was noted that ADS and prior cesarean scars are also risk situations for bladder injury during TVH [41].

Currently, an enlarged uterus with a weight of 300 g or greater is no longer considered an absolute contraindication to minimally invasive surgical approaches, as accumulating evidence has demonstrated their safety, feasibility, and effectiveness in appropriately selected patients. Recent studies have shown that laparoscopic hysterectomy for large uteri can be safely and successfully performed by experienced surgeons. Consequently, uterine size alone should no longer be regarded as the primary determinant in selecting minimally invasive approaches, including laparoscopic hysterectomy and VH [42].

Beyond different surgical techniques, increasing attention has also been directed toward optimizing postoperative tissue healing and recovery in gynecologic surgery in order to increase patient quality of life [43]. Emerging evidence suggested that biologically active adjuncts may enhance tissue regeneration, reduce inflammation, and potentially improve clinical outcomes. However, recent studies have explored adjunctive strategies aimed at improving wound healing, reducing inflammation, enhancing postoperative recovery and patient comfort in gynecological settings [44,45]. Although such interventions were not evaluated in the present study, these developments highlight the broader interest in perioperative optimization and may represent an area for future investigation alongside minimally invasive surgical approaches such as TVH. However, future multicenter studies with long-term follow-up are recommended to validate these findings, exploring also the role of robotic-assisted hysterectomy [42].

In accordance with STROBE recommendations, the limitations of this retrospective design must be acknowledged, including potential selection bias, lack of adjustment for confounding variables, and limited external validity due to the single-surgeon, single-center cohort. The single-surgeon design represents both a strength and a limitation. On one hand, it ensures technical consistency, and on the other hand, it may limit generalizability and introduce operator-dependent bias. Therefore, our results indicate that the prevalence of diffuse ADS among patients undergoing hysterectomy is large, highlighting its significant role in contributing to uterine symptoms that lead to hysterectomy in women over 40 years of age. In a recent large cohort study, an overall ADS incidence of 28.9 per 10,000 women was observed, with the highest incidence observed in women aged between 41 and 45 years [46]. Further, ADS can create diagnostic difficulties, as its imaging features and clinical presentation may resemble malignancy, including abnormal uterine bleeding or uterine enlargement. Interestingly, the use of ultrasonography had substantially improved the management of such cases, our findings being consistent with existing data [44], which does not bypass any socio-cultural environment [47].

In a retrospective analysis, Chen et al. [48] found that 71.8% of patients with ADS presented with marked symptoms. The high prevalence in these cohorts underscores the clinical significance of ADS in uterine pathology and the need for reliable diagnostic tools to enable early detection.

### 4.2. Strengths and Weaknesses of the Study

One of the strengths of our study is represented by the homogenous population and uniformity in the surgical techniques achieved by the same surgeon. Our study describes the TVH technique in a way that facilitates its reproduction and a significant reduction in operative time, intraoperative blood loss, and length of hospital stay, which underlines the economic advantages. Another strength of our study could be explained by the fact that none of our TVH cases needed conversion to TAH, and the data come from a tertiary center, reflecting real experience in clinical practice. Finally, by applying our protocol similarly with the current recommendation, which involves preoperative endometrial biopsy, the suspicion of occult uterine malignancy.

The limitations of our study include its retrospective design and the absence of randomized allocation between surgical approaches. Although the selection of TVH followed predefined clinical and anatomical considerations, including uterine mobility, vaginal accessibility, and surgical feasibility, preferential allocation of patients with more favorable characteristics to the vaginal route may have introduced a degree of selection bias and residual confounding. Additionally, all procedures were performed by a single experienced surgeon, which ensured technical consistency but may limit the generalizability of the findings, reflecting operator-dependent decisions. Further limitations include restrictions related to patient-specific clinical characteristics and the relatively small number of patients with previous surgeries associated with extensive adhesions.

## 5. Conclusions

In patients with enlarged diffuse uterine ADS, TVH with bilateral adnexectomy was associated with younger age, shorter operative time, reduced intraoperative blood loss, and a shorter length hospital stay compared with TAH, without an observed increase in perioperative complications. Our study supports the use of TVH as a feasible surgical approach for selected enlarged diffuse uterine ADS.

## Figures and Tables

**Table 1:** Measures of dispersion and central tendency for continuous variables in TVH and TAH groups.

Characteristics	TVH Group (*n* = 80)	TAH Group (*n* = 80)	*p* Value
Uterine weight (g)			
Mean ± SD	276.48 ± 15.52	278.34 ± 15.70	0.483 *
Median, IQR	278.00, 26.5	278.00, 28.75	
BMI (kg/m^2^)			
Mean ± SD	28.33 ± 4.40	28.68 ± 4.17	
Median, IQR	29.35, 6.23	29.25, 6.03	0.696 **
Parity			
Mean ± SD	1.71 ± 1.19	1.73 ± 1.19	
Median, IQR	2.00, 1.00	2.0, 1.00	0.922 **
Operative time (min)			
Mean ± SD	54.84 ± 11.57	82.26 ± 26.11	
Median, IQR	55.00, 20.50	80.5, 48.25	<0.001 **
Blood loss (mL)			
Mean ± SD	106.69 ± 29.85	136.19 ± 28.02	
Median, IQR	114.50, 48.75	135.00, 40.00	<0.001 **
Hospital stay (days)			
Mean ± SD	3.94 ± 0.80	5.38 ± 1.12	
Median, IQR	4.00, 2.00	5.00, 2.00	<0.001 **

BMI = body mass index; SD = standard deviation; IQR = interquartile range. * Based on Independent Samples *t*-test; ** Based on Mann–Whitney U test.

**Table 2:** Distribution of percentages for categorical variables in patients undergoing TVH and TAH.

Characteristics	TVH Group, *n* (%)	TAH Group, *n* (%)	*p* Value
Medical history	24 (30.00)	27 (33.75)	0.611
Surgical history	22 (27.50)	43 (53.75)	0.001
Intraoperative complications	-	3 (3.75)	-
Postoperative complications	3 (3.75)	11 (13.75)	0.025
Adhesions	7 (8.75)	14 (17.50)	0.101

## Data Availability

All data supporting the findings of this study are included within the article.

## Abbreviations

The following abbreviations are used in this manuscript:

ADS	Adenomyosis
BMI	Body mass index
IQR	Interquartile range
MRI	Magnetic resonance imaging
TVH	Total vaginal hysterectomy
TAH	Total abdominal hysterectomy
SD	Standard deviation
UTI	Urinary tract infection
